# Implementing Policy and Practice Changes to Support Breastfeeding Duration in New York State Communities

**DOI:** 10.5888/pcd21.240003

**Published:** 2024-07-11

**Authors:** Ann Lowenfels, Megan Murphy, Abbie Archibald, Sarah Avellino, Katie Potestio

**Affiliations:** 1 Bureau of Chronic Disease Evaluation and Research, New York State Department of Health, Albany; 2 Bureau of Community Chronic Disease Prevention, New York State Department of Health, Albany

## Abstract

The health benefits of breastfeeding are well-documented, but rates of breastfeeding duration in the US fall below national targets — especially when mothers have less education, have lower incomes, are non-Hispanic Black, or live in nonmetropolitan areas. The Creating Breastfeeding Friendly Communities program was designed to promote breastfeeding and reduce disparities by implementing policy and practice changes in worksites from 2017 through 2023. The purpose of this evaluation was to determine whether the program was effective in increasing breastfeeding supports and addressing disparities. We used a 14-item tool to assess breastfeeding policies and practices at baseline and follow-up at each worksite. We used number of employees to determine worksite size, and we used worksite address to calculate social vulnerability of the community where each site was located and to classify rurality of the county where sites were located. We found significant improvements in the number and quality of breastfeeding supports available at participating worksites (N = 292 at baseline and follow-up). The program successfully reached worksites in socially vulnerable communities. Supports for breastfeeding increased in all worksite subgroups, but they increased less at worksites that were small or rural. The evaluation supports the effectiveness of worksite lactation programs and protective labor laws. Findings suggest that special attention must be given to worksites that are small, located in socially vulnerable communities, or rural counties, to support implementation and reduce disparities.

**Summary**

**What is already known on this topic?**

Breastfeeding duration in the US falls below national targets — especially when mothers have less education, have lower incomes, are non-Hispanic Black, or live in nonmetropolitan areas.

**What is added by this report?**

A worksite lactation program in New York State was associated with significant increases in the number of breastfeeding supports offered. The program reached worksites in communities determined to have a high level of social vulnerability based on socioeconomic status and racial and ethnic minority characteristics. The program was particularly successful in worksites that were large or in urban areas.

**What are the implications for public health practice?**

To support implementation and prevent disparities, lactation support programs should focus on worksites that are small, rural, or in socially vulnerable communities.

## Introduction

For mothers, breastfeeding lowers the risk of chronic diseases such as obesity, diabetes, high blood pressure, and some cancers (1–3). For infants, breastfeeding lowers the risk of obesity, diabetes, asthma, sudden infant death syndrome, and several infectious diseases (4). Some of these benefits may increase with exclusive breastfeeding and longer duration of any breastfeeding (5). To promote breastfeeding in the US, the US Department of Health and Human Services established national targets for exclusive breastfeeding until 6 months and any breastfeeding at 1 year. Breastfeeding rates fall below these targets — especially when mothers have less education, have lower incomes, are non-Hispanic Black, or live in nonmetropolitan areas (6). Because the root causes of these disparities are systemic, effective solutions must include broad-based policy, systems, and environmental changes.

Returning to work creates challenges for breastfeeding employees (7), especially for people who work for smaller employers, have lower-paying jobs, or experience structural racism (8–10). To address these challenges, some breastfeeding promotion strategies focus on worksites. National laws such as the Fair Labor Standards Act require flexible break times for pumping breast milk and private spaces to express milk (11), and state laws such as the New York State Labor Law 206-c require additional accommodations (12). State and national programs help worksites understand, implement, and promote these laws. The State Physical Activity and Nutrition program supported worksites in funded states from September 2018 through September 2023, and the Creating Breastfeeding Friendly Communities (CBFC) program supported worksites in funded communities throughout New York State from February 2017 through June 2023. These kinds of worksite programs can improve supports for breastfeeding (13), increase rates of breastfeeding (14,15), and offer measurable benefits to employers (16,17).

## Purpose and Objectives

The purpose of the CBFC program was to promote breastfeeding and reduce disparities by implementing policy, systems, and environmental changes in multiple settings, including worksites. The program was funded by the New York State Department of Health, with support from the Centers for Disease Control and Prevention (CDC). It was implemented by CBFC grantees, who encouraged worksites to implement evidence-based supports recommended in the CDC Worksite Health Scorecard (18) and required by federal and state labor laws (11,12). A total of 316 worksites agreed to participate in this program. The purpose of the evaluation was to determine whether the program was effective in increasing breastfeeding supports and addressing disparities by answering 3 questions: Did the program help participating worksites implement policy, systems, and environmental changes? Did the program reach worksites that were small or located in socially vulnerable communities or rural counties? Did the program increase breastfeeding supports at worksites that were small or located in socially vulnerable communities or rural counties? Answers to these questions were expected to contribute to the evidence base for worksite breastfeeding promotion programs.

## Intervention Approach

In New York State, 87% of newborns start breastfeeding, but only 23% are exclusively breastfeeding at 6 months (19). To support and promote breastfeeding duration in New York State, the Department of Health used sociodemographic indicators to identify priority communities. The department used a competitive application process to select 6 grantees: 2 county health departments, 3 medical centers/academic institutions, and 1 community-based organization. These grantees had experience working in the priority communities, and they were responsible for implementing the CBFC program from February 1, 2017, through June 30, 2023. Intervention activities included recruiting and assessing worksites, providing training about breastfeeding laws and accommodations to employers and employees, helping worksites strengthen their lactation policies, and encouraging employers to use available tools and resources to support policy implementation. Grantees also developed worksite recognition programs and publicly recognized employers for implementing recommended strategies to improve breastfeeding support. These activities were expected to increase the number of worksites that provide accommodations for breastfeeding employees and contribute to increases in breastfeeding exclusivity and duration in priority communities.

## Evaluation Approach

We used a pre–post quasi-experimental design to determine whether the CBFC program was effective in increasing breastfeeding supports and addressing disparities. We developed a 14-item tool to assess breastfeeding policies, systems, and environmental supports at participating worksites. The tool included 6 validated lactation items from CDC’s Worksite Health ScoreCard (18) and 8 items that focused on state laws and CBFC program goals. CBFC grantees started collecting data in May 2017 and finished in June 2023. Grantees were trained to conduct assessments when they started working with a site (baseline) and when they finished working with a site (follow-up). They completed assessments in an interview format with key staff who were knowledgeable about the worksite’s policies and practices. For each item, they responded yes if the support was in place and no if it was not. After each interview, grantees used SurveyMonkey (www.surveymonkey.com) to submit responses to the New York State Department of Health. This project did not require institutional review board approval because it did not involve human subjects; we used only site-level data.

To measure disparities, we created indicators for worksite size, social vulnerability, and rurality. We collected data on worksite size during the baseline assessment; we categorized worksites as small if they had fewer than 50 employees and large if they had 50 or more employees. For social vulnerability, which refers to the negative effects on communities as a result of external stresses on health, we used the CDC/Agency for Toxic Substances and Disease Registry’s (ATSDR’s) Social Vulnerability Index (SVI) (20). The SVI has 4 themes: socioeconomic status, household characteristics, racial and ethnic minority status/language, and housing type/transportation. We used themes 1 and 3 to calculate measures of socioeconomic status and racial and ethnic minority status for the community in which the worksites were located. For these calculations, we used methods developed and described by CDC/ATSDR (20) to combine data from census tracts into communities. We ranked communities in New York State from highest to lowest social vulnerability. We categorized social vulnerability scores as high if the worksite was in a community in the top 75th percentile for either socioeconomic status or racial and ethnic minority status (ie, the higher the percentile, the greater the negative effect of stresses). Otherwise, we categorized social vulnerability scores as low. We determined rurality according to the 2013 National Center for Health Statistics Urban–Rural Classification Scheme for Counties (21). We classified worksites as rural if they were in a county classified as noncore or micropolitan and urban if they were in a county classified as metropolitan.

We used SAS 9.4 (SAS Institute, Inc) in October 2023 to assess changes from baseline to follow-up; all differences were assessed at a significance level of .05. First, we compared the proportion of worksites responding yes to each support at baseline with the proportion responding yes at follow-up. We used McNemar tests to determine significant differences in these proportions. Next, we scored (range, 0–14) each worksite for the number of supports in place at baseline and follow-up; then we calculated mean scores at baseline and follow-up for all worksites and for worksites according to size, social vulnerability score, and rurality. We used paired-sample *t* tests to compare mean scores at baseline and follow-up. Then we used independent sample *t* tests to examine differences between small and large worksites, worksites with high and low social vulnerability, and rural and urban worksite locations at baseline and follow-up. Finally, we used multiple linear regression to test whether baseline scores, worksite size, social vulnerability, or rurality independently predicted changes in scores from baseline to follow-up.

## Results

Of the 316 worksites that completed baseline assessments, 38% were small, 77% were in socially vulnerable communities, and 38% were in rural counties. Of the 316 worksites, 292 (92%) completed a follow-up assessment. Of the 24 worksites that did not complete follow up assessments, 7 had not finished implementing the program, 8 had discontinued participation, and 9 were lost to follow-up.

We found significant increases in the proportions of worksites implementing all breastfeeding supports from baseline to follow-up (Table 1). For policy supports, the proportion of worksites with comprehensive policies increased from 33% to 88% (a 167% increase) and the percentage with flexible break times for pumping increased from 93% to 97% (4%).

**Table 1:** Breastfeeding Supports in Place at Baseline and Follow-Up Among Selected Worksites in New York State, 2017–2023^a^

Supports	Baseline, no. (%) (N = 292)	Follow-up, no. (%) (N = 292)^b^
**Policy supports**		
Written breastfeeding policy^c^	165 (57)	266 (91)
Comprehensive breastfeeding policy^d^	97 (33)	258 (88)
Paid maternity leave^c^	145 (50)	191 (65)
Flexible break times for pumping^c^^,^ d	271 (93)	283 (97)
**System supports (institutional practices)**		
Provides information about policies and accommodations	97 (33)	249 (85)
Promotes awareness of policies and labor laws^d^	163 (56)	261 (89)
Offers supervisory training	69 (24)	233 (80)
Provides support groups or educational classes^c^	41 (14)	102 (35)
Offers opportunities for support and encouragement	155 (53)	257 (88)
Maintains a list of community resources	45 (15)	245 (84)
**Environmental supports**		
Private space to express milk^c^^,^ d	209 (72)	272 (93)
Private space is convenient, accessible, and has amenities^d^	136 (47)	260 (89)
Access to breast pump^c^	33 (11)	134 (46)
Access to refrigerator	218 (75)	280 (96)

a The authors conducted a baseline assessment and a follow-up assessment in collaboration with the Creating Breastfeeding Friendly Communities program at 292 worksites.

b All changes from baseline to follow-up were significant at the .05 level; determined by McNemar test.

c Supports described in the Centers for Disease Control and Prevention’s Worksite Health ScoreCard lactation module (18).

d Supports include requirements from Section 206-c of the New York State Labor Law (12).

For system supports (institutional practices), the percentage of worksites that promote awareness of policies and labor laws increased from 56% to 89% (a 59% increase) and the percentage that provide breastfeeding support groups or educational classes increased from 14% to 35% (a 150% increase).

For environmental supports, the percentage of worksites that offer private space to express milk increased from 72% to 93% (a 29% increase); the percentage where private space is convenient, accessible, and has amenities increased from 47% to 89% (an 89% increase); and the percentage with access to a breast pump increased from 11% to 46% (a 318% increase).

We found significant increases from baseline to follow-up in the mean number of breastfeeding supports among all worksites and all worksite subgroups (Table 2). We found significant disparities between some subgroup scores at follow-up: the mean number of breastfeeding supports was lower at small (vs large) worksites (10.5 vs 11.7 supports; *P* < .001) and rural (vs urban) worksites (10.6 vs 11.7 supports; *P* < .001). Higher baseline scores, small worksite size, high social vulnerability, and rurality each independently predicted smaller increases in breastfeeding supports from baseline to follow-up (*R* ^2^ = 0.438).

**Table 2:** Mean Number of Breastfeeding Supports (of 14 Maximum) in Place at Baseline and Follow-Up, By Worksite Characteristics, Among Selected Worksites (N = 292) in New York State, 2017–2023^a^

Worksite characteristic	No. of worksites	Baseline, mean (SD)	Follow-up, mean (SD)^b^
**All worksites**	292	6.3 (3.0)	11.3 (2.9)
**Worksite size**			
Small (<50 employees)	115	6.2 (3.2)	10.5 (3.4)
Large (≥50 employees)	177	6.4 (2.9)	11.7 (2.3)
**Social vulnerability score^c^**			
High	219	6.2 (3.1)	11.1 (3.1)
Low	73	6.6 (2.5)	11.8 (1.8)
**Rurality of county in which worksite was located^d^**			
Rural (noncore or micropolitan)	120	6.0 (2.6)	10.6 (3.3)
Urban (metropolitan)	172	6.5 (3.2)	11.7 (2.4)

a The authors conducted a baseline assessment and a follow-up assessment in collaboration with the Creating Breastfeeding Friendly Communities program at 292 worksites.

b Paired *t* tests were used to compare the mean scores at baseline and follow-up for all worksites and for each subgroup of worksites; all differences from baseline to follow-up were significant at the <.001 level.

c Determined by using Centers for Disease Control and Prevention/Agency for Toxic Substances and Disease Registry’s Social Vulnerability Index (20), which includes data on socioeconomic status and racial and ethnic minority status. Scores were categorized as high if the worksite was in a community in the top 75th percentile for either socioeconomic status or racial and ethnic minority status. Otherwise, they were categorized as low.

d Determined by 2013 National Center for Health Statistics Urban–Rural Classification Scheme for Counties (21).

## Implications for Public Health

This evaluation provides support for the effectiveness of worksite lactation programs like CBFC. After participating in the program, worksites reported having more policies, systems, and environmental supports for breastfeeding. This finding highlights the importance of funding lactation support programs and the need to expand initiatives like CDC’s State Physical Activity and Nutrition program (https://www.cdc.gov/nccdphp/dnpao/state-local-programs/span/span-2023.html), which is currently only able to support work in 17 states. A full cost-benefit analysis of worksite lactation programs should be conducted.

This evaluation also demonstrates the importance of supportive labor laws. At baseline, supports required by national labor laws, such as flexible break times for pumping and private space to express milk, were more common than other supports. At follow-up, supports required by state labor laws were also more common than other supports: comprehensive breastfeeding policy, promoting awareness of policies and laws, and offering private space with required amenities. At baseline and follow-up, 2 supports (support groups/educational classes and access to a breast pump) not required by national or state labor laws were less common than other supports, even though they are considered best practices and included in CDC’s Health ScoreCard.

This evaluation shows that CBFC was successful at reaching worksites located in socially vulnerable communities defined on the basis of socioeconomic status and racial and ethnic minority status. This finding highlights the value of identifying priority communities and collaborating with partners who have experience working in them. Supports for breastfeeding increased in all worksite subgroups, but they increased less at worksites that were small or rural. Some supports may have been more difficult to implement in small worksites, such as support groups and educational classes. To support implementation and reduce disparities, special attention must be given to worksites that are small or located in socially vulnerable communities or rural counties.

Our study sample was large and diverse, and we were able to report results by key worksite characteristics, including size, social vulnerability, and rurality. Our evaluation used rigorous methods, which enabled us to generalize our findings about policy, systems, and environmental changes at CBFC worksites. However, our evaluation has several limitations. The design did not include a comparison group, so we were unable to measure the effect of policy changes that occurred during the CBFC program, such as the Providing Urgent Maternal Protections (PUMP) for Nursing Mothers Act (11). Future evaluations should include a comparison group. We used a convenience sample of worksites that were willing to participate in CBFC, which limited our ability to generalize the findings, although the final sample was diverse and large enough for us to conduct subgroup analyses. Survey responses were not objectively verified, but almost half of the survey items had already been validated by others (18). Finally, our evaluation did not collect employee-level data, so we could not determine if site-level changes contributed to increases in breastfeeding initiation and duration. However, this connection has already been demonstrated by others (14,15).

Future programs should prioritize worksites located in socially vulnerable communities, ensure participation among small worksites in rural counties, and be tailored to offer these worksites additional support to promote implementation.
